# Clinically encountered growth phenotypes of tuberculosis-causing bacilli and their *in vitro* study: A review

**DOI:** 10.3389/fcimb.2022.1029111

**Published:** 2022-11-10

**Authors:** Saurabh Mishra, Kohta Saito

**Affiliations:** ^1^ Department of Microbiology and Immunology, Weill Cornell Medicine, New York, NY, United States; ^2^ Department of Medicine, Weill Cornell Medicine, New York, NY, United States

**Keywords:** tuberculosis, mycobacteria, persisters, VBNC, oxidative stress, heterogeneity, differentially detectable

## Abstract

The clinical manifestations of tuberculosis (TB) vary widely in severity, site of infection, and outcomes of treatment—leading to simultaneous efforts to individualize therapy safely and to search for shorter regimens that can be successfully used across the clinical spectrum. In these endeavors, clinicians and researchers alike employ mycobacterial culture in rich media. However, even within the same patient, individual bacilli among the population can exhibit substantial variability in their culturability. Bacilli *in vitro* also demonstrate substantial heterogeneity in replication rate and cultivation requirements, as well as susceptibility to killing by antimicrobials. Understanding parallels in clinical, *ex vivo* and *in vitro* growth phenotype diversity may be key to identifying those phenotypes responsible for treatment failure, relapse, and the reactivation of bacilli that progresses TB infection to disease. This review briefly summarizes the current role of mycobacterial culture in the care of patients with TB and the *ex vivo* evidence of variability in TB culturability. We then discuss current advances in *in vitro* models that study heterogenous subpopulations within a genetically identical bulk culture, with an emphasis on the effect of oxidative stress on bacillary cultivation requirements. The review highlights the complexity that heterogeneity in mycobacterial growth brings to the interpretation of culture in clinical settings and research. It also underscores the intricacies present in the interplay between growth phenotypes and antimicrobial susceptibility. Better understanding of population dynamics and growth requirements over time and space promises to aid both the attempts to individualize TB treatment and to find uniformly effective therapies.

## Introduction

When a human host encounters a member of the *Mycobacterium tuberculosis* complex (MTBC), the potential outcomes range the entire breadth of clinical possibilities—from immediate sterilization, to asymptomatic infection with later progression to active disease, to fulminant tuberculosis (TB) disease. The necessary duration of therapy for relapse-free cure also varies greatly, although months of multi-drug therapy is standard. At the most simplistic level, this is a matter of whether the bacillus can replicate in its local microenvironment and, if it is unable to replicate, whether it can subsequently regain that ability when stresses are removed or altered. If disease state heterogeneity in the human population reflects the heterogeneity of *in vivo* organism replication, then it is tantalizing to use *ex vivo* and *in vitro* culturability—and resuscitation of culturability—to draw clinically relevant conclusions regarding drug efficacy and treatment outcomes. In this review, we briefly examine the role of mycobacterial culture in clinical care and clinical research, and then explore how *ex vivo* work connects these clinical observations. We then survey the conditions and cellular mechanisms driving heterogeneity in growth *in vitro*, emphasizing the role of oxidative stress. In so doing, we endeavor to show that while it would be ideal to make culture obsolete in the clinic, there is still much to be gained at the bedside from better understanding of the wide variability in growth speed and replication requirements of stressed MTBC organisms at the bench.

## Clinical use of mycobacterial culture

**Figure 1:**
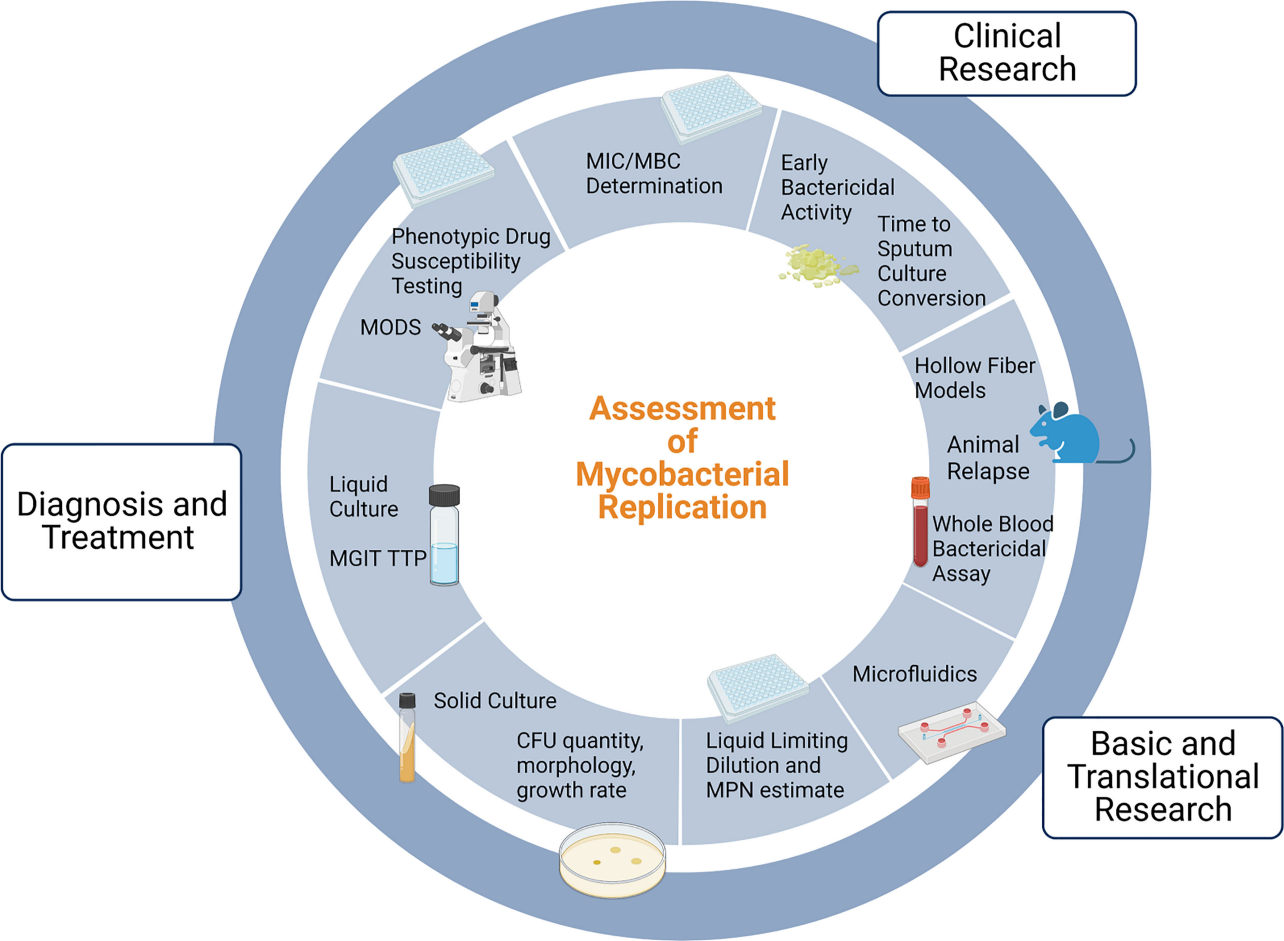
Uses of and methods for assessment of mycobacterial replication. Note that drug susceptibility testing incorporates clinical and *in vivo* factors in addition to MIC testing and is thus distinct. MGIT: Mycobacterial Growth Indicator Tube; TTP: time to positivity; MODS: Microscopic Observation Drug Susceptibility; MIC: minimum inhibitory concentration; MBC: minimum bactericidal concentration; MPN: Most Probable Number; CFU: Colony Forming Units. Adapted from “Gut-Brain Axis Regulators”, by BioRender.com (2022). Retrieved from https://app.biorender.com/biorender-templates.

**Figure 1** provides an overview of the uses and methods for assessment of mycobacterial replication. The MTBC is a group of genetically related mycobacterium species that can cause TB, and includes *Mycobacterium tuberculosis* (Mtb), *Mycobacterium africanum*, and *Mycobacterium bovis (M. bovis)*, among others. In the clinical setting, cultivation of MTBC bacilli serves three main purposes: diagnosis of TB disease, monitoring response to therapy, and phenotypic drug susceptibility testing. Culture positivity and conversion to negativity are often used by clinicians to help personalize TB treatment, and culture-based endpoints are used in clinical trials as surrogate markers of durable cure or treatment failure. Here we will briefly describe the vital but imperfect role of culture in the clinic and in clinical research.

### Diagnosis and selection of treatment

Mycobacterial culture is currently the laboratory gold standard for the microbiologic diagnosis of TB disease despite improvements in molecular methods of mycobacterial detection (Lewinsohn et al., 2017). Growth of Mtb or other MTBC bacilli from a patient produced sample can secure the diagnosis, however, culture yields can be highly variable and affected by bacillary load, specimen type, and specimen processing. As an example, MTBC organisms are isolated from less than 75% of infants and 50% of children with pulmonary TB as diagnosed by clinical criteria—the numbers are likely even lower for many extrapulmonary forms of TB (Graham et al., 2015; DiNardo et al., 2016; Thomas, 2017; Baker, 2020). Liquid culture methods have generally been more sensitive than solid culture methods—88-90% vs 76% in one meta-analysis—and have a shorter time to detection. The same meta-analysis demonstrated a drop in sensitivity of liquid culture in AFB sputum that were negative for acid fast bacilli (AFB) on smear to ≤ 80% (Cruciani et al., 2004). Innovations in liquid culture readout, such as the Mycobacteria Growth Indicator Tube (MGIT) that contains an oxygen-quenched fluorochrome which fluoresces when growing mycobacteria consume oxygen, among others, facilitate diagnosis and can be implemented in a simple, safe, and automated or semi-automated fashion (Palaci et al., 1996; Pfyffer et al., 1997; Heifets et al., 2000). The use of this fluorescence indicator mitigates the downsides of previously used radiometric readouts for liquid culture that are expensive and produce radioactive waste, while maintaining similar sensitivity for Mtb detection (Pfyffer et al., 1997; Cruciani et al., 2004). However, liquid cultures are more prone to contamination with other bacteria; therefore, current guidelines endorsed by the CDC recommend using both solid and liquid culture systems (Ichiyama et al., 1993; Lewinsohn et al., 2017). Liquid culture systems require addition of an antimicrobial cocktail to reduce contamination rates, although rates still ranged in the meta-analysis from 3.8 to 16.6% for MGIT (Cruciani et al., 2004). The MGIT system includes polymyxin B, amphotericin B, nalidixic acid, trimethoprim, and azlocillin (PANTA) in the media. This cannot, however, mitigate contamination with species or isolates that are resistant to the cocktail (Cornfield et al., 1997). MGIT also supports growth of many non-tuberculous mycobacteria. Given these known variabilities in culture yield, physicians must ultimately rely on clinical judgement if there is a high suspicion of active TB in a patient, even with negative culture results.

To maximize yield, current CDC guidelines recommend three sputum smears for assessment of AFB by microscopy as well as sending all collected sputum for mycobacterial culture (Lewinsohn et al., 2017). This is in line with data showing that obtaining multiple cultures improves sensitivity, with the largest improvement occurring with a second specimen and smaller incremental gains thereafter (Blair et al., 1976; Levy et al., 1989; Cascina et al., 2000; Harvell et al., 2000) It is important to note that given the laboratory requirements and costs of performing mycobacterial culture, it is not always feasible to obtain culture for TB diagnosis, and the WHO has reviewed and endorsed several rapid molecular methods as initial diagnostic tests for TB rather than smear microscopy and culture (2021).

Each patient merits careful consideration when being evaluated for TB, as host characteristics can profoundly affect the diagnostic approach. For example, in people with HIV, sputum that is smear-negative but culture-positive is common—one study out of Thailand and Vietnam found that three MGIT cultures missed only 3 of 126 patients with pulmonary TB, whereas three sputum smears missed 79 (Monkongdee et al., 2009). Interestingly, that study also found that a single liquid culture yielded a TB diagnosis in a similar number of patients as three sputa on solid media. As noted in CDC guidelines, in HIV-related TB, enlarged lymph nodes can often yield a diagnosis when aspirated, and in severely immunocompromised patients’ urine and blood cultures can also yield mycobacteria (Panel on Guidelines, 2022). In children, as previously noted, microbiologic confirmation of TB is difficult, and even in pulmonary TB expectorated sputum can be difficult or impossible for young children to produce. Again, culture of other specimens is considered and has been studied, including sampling of gastric fluid, nasopharyngeal aspirates, stool, and urine. One recent study of children under 5 who had symptoms of TB found that combinations of tests and repetition of tests led to increased yield—and found that some combinations of non-invasive tests could perform similarly to more invasive procedures such as gastric aspiration or induced sputum (Song et al., 2021). In that study, MGIT was more sensitive than the rapid molecular diagnostic test Xpert, but Xpert identified some children who remained MGIT negative. However, the overall rate of microbiologic diagnosis was still low at 10%, and it is unknown how many children actually had TB given the information provided in the report (Starke and Cruz, 2021). Thus, despite current culture techniques remaining the laboratory gold standard, better and scalable diagnostics, whether or not they are culture-based, are desperately needed.

If TB exposure is diagnosed by non-culture methods, negative culture results can help define where along the clinical spectrum an individual lies. This is clinically relevant, as treatment selection can be directly impacted. For example, in considering treatment for TB infection, some patients first undergo sputum cultures to assess for TB disease—lest a patient inappropriately receive monotherapy for TB disease that would select for drug resistance. For pulmonary TB disease, growing evidence suggests that some patients who are diagnosed clinically but cannot be confirmed microbiologically can achieve durable cure with therapy shorter than the current 6-month standard. In adults, the Infectious Diseases Society of America guidelines suggest that certain patients with culture-negative pulmonary tuberculosis can be safely treated with a 4-month regimen based on a systematic review of studies out of Hong Kong, Arkansas, and Singapore (Sotgiu et al., 2016). As alluded to above, many children belong to this kind of ‘paucibacillary’ group, and the recent SHINE trial demonstrated non-inferiority of a 4-month treatment regimen in drug-susceptible, smear-negative, non-cavitary TB. Of 1204 patients in that trial, only 146 had a positive Mtb culture (Turkova et al., 2022). Less clear is how to approach patients in the converse situation: with minimal-to-no symptoms but found to have microbiologic confirmation of TB disease in sputum. Such subclinical TB is increasingly recognized by studies using culture and molecular diagnostics, but questions remain regarding the clinical significance of this finding—including rates of transmission from this population, the true proportion of patients who progress to more severe disease, and what, if any, therapy is required (Frascella et al., 2021; Kendall et al., 2021; Wong, 2021).

Phenotypic drug susceptibility testing (DST) can be performed when an isolate is recovered *via* culture. This remains the reference standard DST for many TB drugs, especially as new or repurposed drugs are developed for which resistance mechanisms are unknown or not fully defined. However, this method takes resources and time, and efforts have been made to make more accessible culture-based drug susceptibility tests. For example, the Microscopic Observation Drug Susceptibility (MODS) assay takes advantage of the faster growth of MTBC organisms in liquid culture and uses an inverted microscope to rapidly detect growth. The cording pattern of mycobacterial growth allows for relatively facile visualization. If the bacilli grow in the presence of drug, resistance is detected. While not FDA approved in the US, studies have found it may have utility as a rapid, inexpensive, and reliable alternative to other methods of drug susceptibility testing, despite the need for appropriate training and specific supplies (Caviedes et al., 2000; Moore et al., 2004; Moore et al., 2006; Fitzwater et al., 2010; Minion et al., 2010; Shah et al., 2011; Alcántara et al., 2020). Very rapid molecular methods of detecting drug resistance, not reviewed here, are quickly improving and expanding available tests (Lewinsohn et al., 2017; WHO, 2021). Still, the advantages of phenotypic DST in the changeable landscape of drug resistance and therapy make the development of widely-accessible, fast, and clinically-applicable phenotypic DST necessary. Of note, phenotypic DST can be designed to assess raw minimum inhibitory concentrations (MIC) and not simply whether an Mtb isolate is susceptible based on a single breakpoint value. Intriguingly, in one study, higher pretreatment MIC values for isoniazid (INH) or rifampin that were still below the resistance breakpoint were associated with a greater risk of relapse than lower values, even after adjusting for other risk factors (Colangeli et al., 2018; Rubin, 2018). These kinds of findings may eventually allow for better personalization of care—or provide strategies for globally reducing treatment failure and relapse.

### Monitoring during therapy

In TB disease, care often needs to be individualized based on clinical response and the tolerability of drug regimens. Bacillary load and response to TB chemotherapy has been monitored for decades by serially quantifying the number of colony forming units (CFU) of Mtb that grow on a solid medium from sputum. While CFU ideally arise from a single bacillus for accurate quantification, the clumpy nature of these organisms ensures at least some clumps even in well dispersed cultures (Fenner et al., 1949; Stewart et al., 1957; Diacon and Donald, 2014). Growth of a colony simultaneously allows counting *via* the naked eye as well as proof of viability of the progenitor(s) of the colony. However, as we will explore further, the CFU may frequently undercount the number of viable mycobacteria or be falsely reassuring when negative, especially during therapy.

In current guidelines, treatment failure in drug susceptible TB is defined as Mtb growth in any form of culture after 4 months (or 5 months in WHO guidelines) of appropriate therapy (Sotgiu et al., 2016). Sputum cultures are checked monthly at a minimum until two consecutive cultures are negative (Sotgiu et al., 2016). Earlier indicators of treatment failure and relapse are few. Checking whether sputum cultures grow Mtb at 2 months (the usual end of the intensive phase of treatment) is recommended, and in current CDC guidelines growth of Mtb at 2 months is one criterion for which to consider extending the continuation phase of therapy by several months (Sotgiu et al., 2016; WHO, 2022). However, while persistent positive cultures are a risk factor for failure, the sensitivity of this test in detecting relapse is overall poor—one systematic review and meta-analysis found that the pooled sensitivity for 2-month sputum culture to predict relapse was 40% (Horne et al., 2010). The WHO does not currently endorse extension of either the intensive or continuation phase based on growth at 2 months due to the modest benefit in relapse reduction (Horne et al., 2010; Phillips et al., 2016; Romanowski et al., 2019; WHO, 2022). Culture conversion at 2 months also does not allow for shortening of current standard therapy below 6 months without a significant increase in relapse rates (Johnson et al., 2009). Of note, the original studies correlating 2-month culture status with risk of relapse were performed using traditional solid media, which as previously noted are generally less sensitive than liquid cultures (Johnson et al., 2009). Given these known limitations, culture maintains an important role in treatment monitoring, but the interpretation of results must be individualized and reflect nuanced understanding of the test characteristics.

Time to positivity (TTP) of growth in liquid culture detection systems, also reported as time to detection (TTD) in the literature, has been studied as a marker of risk of relapse both before and during treatment. A short TTP correlates with higher bacillary burden by CFU, and lack of an increase in TTP with therapy may reflect a poor response. TTP is likely affected in individual samples by factors aside from pure bacillary burden, and interestingly one of the early studies found several patients with TTP < 20 hours but whose sputum AFB smears were negative (Epstein et al., 1998; Pheiffer et al., 2008; Hesseling et al., 2010; Bark et al., 2012; Olaru et al., 2014). During TB treatment, the relationship between TTP and CFU is more complex and changes over time—it has been demonstrated that even at the same CFU, TTP becomes longer if the sample is derived from later in treatment. The authors of that study hypothesize this is due to a subpopulation of bacilli that grows and is detected in the liquid culture but is not recovered as CFU (Bowness et al., 2015). This phenotypic state of mycobacteria is reviewed in depth further below.

For drug-resistant TB, using culture in treatment monitoring and in detecting treatment failure or development of drug resistance remains a key recommendation (Nahid et al., 2019; WHO, 2019). While guidelines have been in rapid flux due to the availability of new drugs and regimens in MDR/XDR-TB, the duration of both the intensive and continuation phases of the regimen is often personalized and can be anchored to the time of sputum conversion or the persistence of positive cultures (Nahid et al., 2019). Even in standardized, shorter treatments like the bedaquiline, pretomanid, and linezolid (BPaL) regimen, sputum culture conversion is monitored and re-assessment of phenotypic drug resistance performed in those with delayed response. It should be noted that in most non-tuberculous mycobacterial pulmonary infections, treatment durations are tied to when sputum cultures convert to consistently negative (Daley et al., 2020).

The relationship between persistence of positive culture and infectivity is another debated topic that may have a profound clinical impact when it comes to isolation and public health requirements. While it has been demonstrated that patients with smear-negative but culture-positive sputa can transmit TB to some degree, and that patients can remain culture positive for weeks to months into treatment, the degree to which treated, culture-positive patients can transmit is not clear (Behr et al., 1999; Hernandez-Garduno et al., 2004; Tostmann et al., 2008; Fitzwater et al., 2010; Asadi et al., 2022). Using the TTP in liquid culture has also been proposed as a potential correlate of infectiousness to guide isolation and contact tracing requirements, and one cohort study found a TTD < 9 days in an index case was associated with an increased transmission risk (Ritchie et al., 2007; O'Shea et al., 2014). Infectiousness likely depends on several other factors beyond culturability, such as mycobacterial fitness and relative ability to generate infectious aerosols, and the public health response requires careful consideration of further variables such as the costs of isolation and exhaustive contact tracing.

### Clinical evaluation of new treatment regimens

Quantifying early change in CFU counts was recognized as a way of evaluating new drugs or regimens since the 1950s, and was more formalized in a 1980 study in which 27 TB drugs and regimens were compared for their effect on sputum Mtb CFU numbers over a 2 to 14 day period (Jindani et al., 1980). The methodology is meant to characterize the early bactericidal activity (EBA) of drugs and regimens, and has been described in guidance from both the U.S. Food and Drug Administration and European Medicines Agency as endpoints during early development of new TB therapies (FDA, 2013; Agency, 2017). EBA studies can evaluate the short-term ability of a single agent to kill mycobacteria during active TB, as well as allow for dose ranging and monitoring for short-term toxicities (Diacon and Donald, 2014). The importance of such trials should not be overlooked. However, there are critical limitations. Some clinically proven drugs have modest to no EBA in the first 2 days, such as rifampin, pyrazinamide, and bedaquiline (Jindani et al., 2003; Sirgel et al., 2005; Rustomjee et al., 2008). EBA results may be discordant with the relative ability to sterilize lesions; even dramatic decreases in actively replicating mycobacteria may not accurately predict a drug’s effect on non-replicating or hypometabolic mycobacteria or on the risk of clinical relapse. Interestingly, one EBA trial that used the first-line TB drug INH to optimize early EBA methodology noted a 2-fold greater rate of decline in CFU than the rate of decline in bacterial load as quantified by AFB smear microscopy, thought due to continued visualization of dead organisms (Hafner et al., 1997). While many of these bacilli may truly be dead, some proportion may be viable but not culturable on standard solid media—this phenomenon is discussed in depth in the next section.

Other culture-based, surrogate assessments of the efficacy of therapies have also been used. Time to sputum culture conversion to no growth, especially at the 2-month mark, has been used both clinically and for evaluation of new TB treatments (Wallis et al., 2009; Wallis et al., 2010). Like its clinical utility in predicting relapse, the information that time to sputum culture conversion provides for new regimens is nuanced and must be interpreted carefully (Wallis et al., 2013; Lanoix et al., 2015). TTP in liquid culture has also been studied as a surrogate measure of EBA and in other clinical trials (Diacon et al., 2010; Weiner et al., 2010). All of these culture-based measures may be influenced by geographic or regional differences across sites as well (Sirgel et al., 2001; Mac Kenzie et al., 2011; Bark et al., 2014).

The fluoroquinolones provide a stark reminder of the potential pitfalls in using EBA and time to sputum culture conversion as surrogate measures of treatment efficacy. ReMOX TB, OFLOTUB, and RIFAQUIN were three randomized controlled trials designed to test the hypothesis that inclusion of fluoroquinolones could reduce treatment duration to 4 months instead of the standard 6 months for drug-susceptible TB, and were supported by earlier data that sputum culture conversion to negative at 2 months was improved by fluoroquinolones as well as strong EBA activity (Gillespie et al., 2014; Jindani et al., 2014; Merle et al., 2014; Lanoix et al., 2015). While all three trials confirmed some improvement in time to culture conversion, none could show clinical noninferiority to 6 months of standard treatment. A more recent study using a regimen combining a fluoroquinolone with rifapentine (instead of rifampin) was able to safely shorten treatment to 4 months in addition to demonstrating the reduction in time to culture conversion as compared to standard therapy (Dorman et al., 2021). However, a separate 4-month rifapentine arm without a fluoroquinolone similarly reduced the time to culture conversion but was clinically inferior to the 6-month treatment arm. That trial simultaneously demonstrated that “there is no magic with 6 months of therapy” and that time to sputum culture conversion can be an inadequate early biomarker in predicting relapse-free cure of new regimens (Rubin and Mizrahi, 2021). The limitations of EBA trials and time to culture conversion as surrogates for durable cure have led to the search for other, non-culture-based early markers to speed evaluation of experimental drug treatments. For example, a recent study utilized PET/CT lung imaging at day 14 of treatment and demonstrated that the ReMOX trial regimen was no better than standard therapy in reducing lesion size or inflammation, which was consistent with the subsequent failure of the trial regimen in shortening treatment duration. This method, though expensive, integrates complex host data and shows promise as a potential way of evaluating early drug or drug combination efficacy (Xie et al., 2021). This, and other methods of treatment monitoring and outcome measures, including those interrogating host characteristics and non-culture, molecular methods of mycobacterial load assessment have been recently reviewed (Heyckendorf et al., 2022).

## *Ex vivo* heterogeneity in culturability

What accounts for the variable utility of mycobacterial culture in diagnosis, treatment monitoring, and as surrogate endpoints for treatment success? Although a myriad of host factors, such as ability to cough, relative achievable TB drug levels, adherence to treatment and baseline immune status, play a role in the limitations of detecting Mtb in culture, if we restrict our view to the perspective and experience of the bacillus, there are at least three contributing and interrelated factors: from where in the body we are sampling, the mycobacterial load in that area, and how we culture. In this section, we review the evidence of heterogeneity in growth phenotypes present within populations of mycobacteria taken from patients and argue that the limitations of sputum culture in understanding TB are due in part to a current inability for standard sputum culture to appreciate this diversity.

### What we are sampling

The human body exposed to Mtb can be a sterilizing environment, a medium that allows for survival but minimal-to-no growth, or a growth permissive space, and this property can change over time and space within the same person. Since before the mid-1900s, the variability both among TB lesions and in the ability to grow Mtb from the varied lesions has been well described. In the introduction to his summary and review of the histopathology and microbiology of human lung tissue from patients with TB in the 1940s, Georges Canetti writes:

“Consider the bacillus in the lesion, experiencing such different fates in various foci of the same patient, and the same fate in widely different patients; destroyed in a certain histologic reaction and thriving in another nearby; swarming not by virtue of some mysterious force but simply because it is situated at a site from which swarming is possible (on the surface of a canalicular system); growing rapidly in certain necrotic areas and poorly in others; finding a proper environment only in certain tissues…”

He goes on to review many forms of lesions and the ability to cultivate mycobacteria from them—for example, he summarizes that 57 of 115 caseous or partially calcified lesions are sterile on egg media, whereas four-fifths of calcified lesions are sterile, and 115 of 134 completely sclerotic lesions did not recover mycobacteria on culture (Canetti, 1955). Later work studying resected lung tissue of patients with TB demonstrated a relationship between the type of lung lesion, drug susceptibility of bacilli recovered from the lesion, and the time required for cultivation of those bacilli ex vivo. Of lesions in communication with a bronchus, 85% yielded tubercle bacilli and all but one grew within 8 weeks of incubation. 70% demonstrated drug resistance. In contrast, only 44% of the cavities closed off from bronchi produced positive cultures and required 3-10 *months* of incubation. Only one of these cultures (17%) demonstrated *ex vivo* drug resistance. After growth in culture, these bacilli appeared morphologically normal, grew normally, and produced disease in animals (Loring et al., 1955; Bloom and McKinney, 1999). In other words, even within the same patient, the local microenvironment is extremely diverse and has a profound impact on a bacterium’s ability to survive the immune response and antibiotics as well as its replication rate *in vivo* and its cultivation requirements *ex vivo*.

Much progress has since been made in understanding highly complex diversity in environmental landscape and bacterial phenotype. For example, the caseous core of a necrotic granuloma has been studied with respect to available carbon sources, relative oxygenation, pH, iron availability, variances in distribution of chemotherapy, and host cell types present—and the relative effect on Mtb growth and phenotypic tolerance to antibiotics (Lenaerts et al., 2015; Sarathy and Dartois, 2020). A link between growth rate and drug susceptibility has long been observed, and lack of replication has been used to explain the proportion of bacilli that are able to survive antibiotic exposure when their genetically identical brethren are killed. The true relationship between replication and drug susceptibility is more complex, nuanced, and specific to the drug and the organism, as evidenced by observed dissociations between drug survival and growth rate or replication *in vitro* (Balaban et al., 2013; Wakamoto et al., 2013; Manina et al., 2015; Zhu et al., 2018). This is explored in further detail when we discuss *in vitro* studies in the manuscript. The population of bacteria that survive antibiotics for prolonged periods without classic genetically encoded resistance are called persisters, but the term thus encompasses a diverse spectrum of phenotypes with respect to metabolic activity and replicative capacity (Gold and Nathan, 2017). The phenomenon of relatively prolonged survival in the face of antibiotics—and the evolving terms used to describe forms of this phenomena—are critical to framing research questions and priorities and have been reviewed elsewhere (Balaban et al., 2019; Schrader et al., 2020). Rapid killing of the diverse array of persisters can likely reduce relapse risk with shorter therapies as well as more efficiently treat latent TB infection, but despite major advances these populations still require better characterization (Dartois and Rubin, 2022). The sputum culture is limiting not only as a representative sample of the numbers of viable bacilli in the body, but may be biased as a relative representation of the spectrum of phenotypes that exist deeper in tissues disconnected from larger airways. Sputum cultures may also be biased by the relative access to and susceptibility of those bacilli to treatment. It should be noted here that efforts to more fully characterize AFB found in sputa have found greater diversity than classically appreciated, as will be described below.

The situation is further complicated when considering the myriad environments in which TB disease can manifest outside the lung—the pleural space, in lymph nodes, the liver, the brain, etc—and the variable ease in obtaining useful culture. For example, in pleural TB, the yield of pleural fluid culture, which requires a thoracentesis, is variable but typically less than 30%, with the yield increasing in patients with HIV (Gil et al., 1995; Gopi et al., 2007; von Groote-Bidlingmaier et al., 2013). As with sputum, the yield improves by using liquid culture systems, but interestingly does not improve with increasing volume of pleural fluid (Luzze et al., 2001; Gopi et al., 2007; von Groote-Bidlingmaier et al., 2013). One interpretation of these findings, as hypothesized by the authors who tested Mtb recovery from differing pleural fluid volumes, is that the immune system produces a dichotomous result—either it will clear the pleural fluid completely or incompletely—and that patients with HIV are more commonly impaired in this pleural fluid clearance function, leading to increased culture yields (von Groote-Bidlingmaier et al., 2013). An alternative explanation is that the viable bacilli relatively sparsely found within the pleural space have specific growth requirements, and the need for such requirements is influenced by the immune system. The increased yield of liquid over solid culture already implies differential growth requirements for a subset of bacteria. Further support for this hypothesis comes from two studies which demonstrated that bedside inoculation of pleural fluid into liquid media as compared to later laboratory inoculation increased yield—suggesting yet another subpopulation of bacteria with specific cultivation needs (Maartens and Bateman, 1991; Augustine et al., 1999). Such variabilities in growth requirements are further explored in the next section.

### How we are culturing

The practicalities of TB sample preparation ensure that quantification of viable *ex vivo* samples cannot be assumed equivalent to the number of culturable bacilli that existed *in situ*—even before they are placed in artificial media. Yield is affected by known factors, such as procedures to decontaminate sputa of non-mycobacterial organisms and room temperature storage (Damato et al., 1983; Paramasivan et al., 1983). The effect of refrigeration and freezing on culture yield of smear-positive sputa has been examined and generally show no significant loss of CFU; however, these studies did note increase in time to positivity of BacT/BACTEC liquid cultures in the same conditions (Tessema et al., 2011; Kolwijck et al., 2013). Sputum processing therefore alters phenotypic growth properties even when there is no apparent change in those bacilli robust enough to form CFU. Theoretically, this may render some bacillary subpopulations incapable of growing in culture and affect yield in paucibacillary cases.

Following such processing, the culture media and environment used in culture-based quantification also affects yield, as has already been touched upon. It has been well described that the sensitivity, specificity, and rapidity of growth in clinical culture systems are all affected by the type of culture media employed. For example, comparison of Middlebrook 7H12 broth, Lowenstein-Jensen, Middlebrook 7H10, and Middlebrook S7H11 media found variable recovery of mycobacteria from smear-negative patient sputa, ranging from 52.1% to 71.8% (Morgan et al., 1983). Efforts have been made to further optimize media composition, outgrowth environmental parameters, such as oxygen tension, and method of visualization, to speed growth and shorten time to detection (Ghodbane et al., 2014; Asmar et al., 2015; Ghodbane et al., 2015).

Recently, studies have found that some Mtb can only grow in liquid limiting dilution with or without supplementation with spent Mtb culture media or resuscitation promoting factors (Rpfs) (Biketov et al., 2000; Shleeva et al., 2002; Kana et al., 2008; Mukamolova et al., 2010; Nikitushkin et al., 2015; Chengalroyen et al., 2016; Dusthackeer et al., 2019). As summarized in the table, such differentially culturable (DC) or differentially detectable (DD) Mtb have been recovered in different *in vitro* models as well as in patient sputa before and during treatment to varying degrees, and their quantity relative to CFU can be orders of magnitude higher. For those DD Mtb that are recovered by liquid limiting dilution, the population size can be estimated using the most probable number (MPN) method, which leverages technical replicates of dilution to extinction to calculate the original, undiluted concentration of viable organisms (Jarvis et al., 2010). While a review of *in vivo* animal models is beyond the scope of this review, it must be mentioned that the phenomena has been recognized for decades in mice who received treatment until bacilli could no longer be recovered in culture, but later relapsed off therapy (McCune et al., 1966). It should also be noted that in the literature, and in other organisms, another often used term is “viable but non-culturable” (VBNC) for similar phenotypes. The basic requirement of these terms is that they define a bacterial state in which the organism does not replicate on traditionally growth-supportive media but is found viable by another method, whether that method is based on resuscitation and return of culturability. A related but distinct phrase is “non-growing but metabolically active” (NGMA), which places focus on bacteria that are not replicating but shows evidence of metabolic activity at a single-cell level and does not require proof of viability (Manina and McKinney, 2013). For brevity and because we are focusing on growth phenotypes, in Mtb we will generally be using the term “DD Mtb,” and in reference to literature from other organisms the term “VBNC,” for the remainder of this review.

It is interesting that some DD Mtb phenotypes require Rpfs for resuscitation, but not others. Rpfs were first discovered in *Micrococcus luteus* as a bacterial equivalent of a cytokine, as it is released extracellularly (Mukamolova et al., 1998). Since then, Rpfs have been identified and characterized in bacteria with high guanine and cytosine content in their genomes, including *Corynebacterium glutamicum* and *Streptomyces* spp (Mukamolova et al., 1998; Mukamolova et al., 2002). Rpfs have been found to resuscitate cells from stress conditions such as prolonged stationary phase, residence in murine peritoneal macrophages, an *in vitro* hypoxia model and, as above, in sputum of patients with TB (Biketov et al., 2000; Shleeva et al., 2002; Chengalroyen et al., 2016; Dusthackeer et al., 2019). Mtb encodes five paralogues of Rpf (Kana et al., 2008). Structurally, Rpfs are similar to lysozyme and lytic transglycosylases (Nikitushkin et al., 2015) and are considered to be important determinants of TB pathogenesis (Rosser et al., 2017). The exact mechanisms of resuscitation with Rpfs and with spent culture filtrate, and why certain phenotypic states are stimulated to divide in their presence, are not fully elucidated but remain an important area of study (Gordhan et al., 2021).

Whether these methods of improving culture yield by finding DD Mtb can meaningfully improve rates of diagnosis and whether the more accurate quantification of mycobacterial load in some patients is clinically useful is being studied. It remains unclear and debated (Walter et al., 2018). McAulay et al. reported that the percentage of patients with DD Mtb in their sputum samples increased from 21% pretreatment to 69% after treatment with isoniazid, rifampin, pyrazinamide, and ethambutol (HRZE) (McAulay et al., 2018). Interestingly, one recent study evaluated for DD Mtb at the end of therapy for drug-susceptible TB *via* bronchoscopy and found that 5 of 41 patients had viable TB. Two of these patients relapsed within one year (Beltran et al., 2020). The relevance of these findings requires further evaluation but suggests that study of this subpopulation yet holds promise.

## Mechanisms underlying heterogeneity in culturability

**Figure 2:**
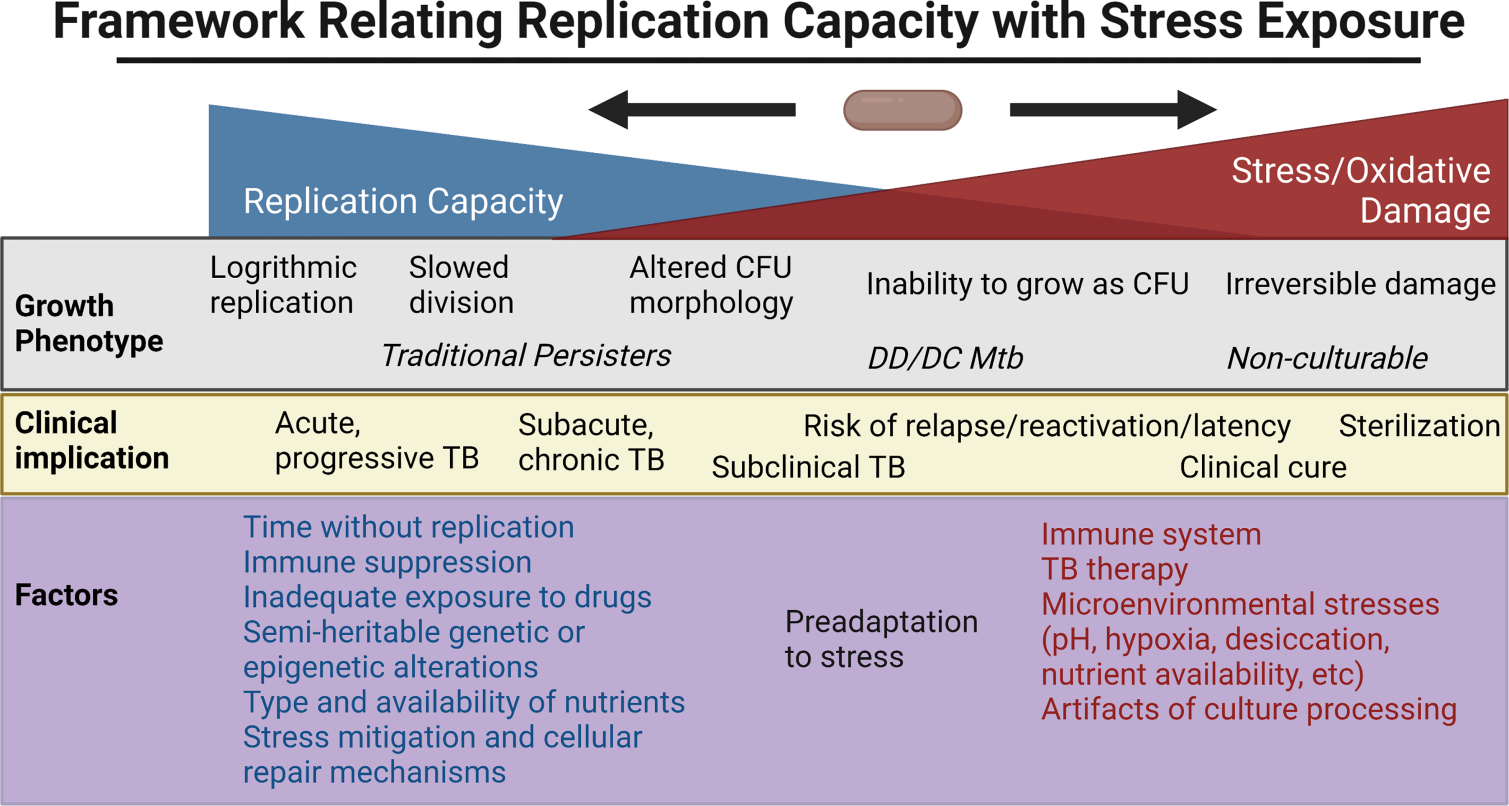
Framework for the relationship between replication capacity and stress/oxidative damage, with the related *in vitro* growth phenotype and *in vivo* clinical implication placed along the spectrum. Factors favoring increased replication capacity and increased stress are listed below. Created with BioRender.com.

The above *ex vivo* studies have shown a broad range of growth phenotypes that MTBC organisms enter as defined by their rate of recovery and growth, need for Rpfs, and resuscitation in liquid but not solid culture media. Clinical outcomes have mirrored this broad range and rate of culturability within the human host. Here, we review some of the extensive efforts to probe this heterogeneity *in vitro*, with a focus on the relationship between reactive oxygen species (ROS), adaptations to cope with oxidative stress, genetic/epigenetic factors and cell division. **Figure 2** places in a framework the interplay between stress, bacterial replication, and its clinical implications. Note that the rich, important literature regarding animal models will not be reviewed here but has been reviewed elsewhere (Fonseca et al., 2017; Zhan et al., 2017).

### Extent of stress and cellular damage alters cultivability requirements

Damage caused by stress can be both oxidative and non-oxidative and can occur either during exposure to stress or during the recovery phase. The **box** inset provides an overview of reduction-oxidation balance in mycobacteria, the way mycobacteria attempt to maintain it, and the damage perturbations to it can cause. Studies in diverse bacterial species have shown that the extent of oxidative stress and damage can affect the cultivation requirements of cells to different levels. High oxygen tension during aerobic growth conditions in microaerophilic *Campylobacter jejuni* causes increases in ROS and in lipid and protein oxidation, and induces formation of coccoidal VBNC cells (Oh et al., 2015). Application of a density-gradient centrifugation technique in *E. coli* found that culturable and non-culturable cells can be separated into two distinct populations, and that proteins in non-culturable cells had increased carbonylation—a hallmark of oxidative damage—in stationary-phase (Desnues et al., 2003) and in heat-stressed cells (Bruhn-Olszewska et al., 2018). Nonthermal plasma (NTP) technology in *Staphylococcus aureus* as an alternative to thermal pasteurization has been shown to cause oxidative stress and induce growth and metabolism quiescence leading to entry into the VBNC state (Liao et al., 2020). A study in *V. parahaemolyticus* further characterized 2 subtypes of VBNC populations, P1 and P2, which are induced by nutrient restriction (PBS starvation) at low temperature 6–8˚C over a period of 50 days (Wagley et al., 2021). 90% of the cells formed a P1 population, defined as small coccoid shaped bacteria that could be resuscitated for up to 14 days. 10% were a P2 population which consisted of bacteria that were large coccoids in shape and could be resuscitated for up to 50 days. While both types of VBNC populations could be resuscitated when subjected to an increase in temperature, the proportion of bacteria which recovered was higher in P2 (100%) as compared to P1 (14%). Proteome analysis of these populations revealed upregulation of the gene encoding lactate dehydrogenase (*lldD*), and an *lldD* deletion mutant entered the VBNC state earlier than the wild-type strain. Addition of sodium lactate resuscitated the VBNC population under conditions when thermal shift methods alone were not adequate. Hong et al. showed that diverse stresses like nalidixic acid, trimethoprim, ampicillin, and heat can induce ROS formation in *E. coli*, that ROS can increase even after the removal of stress, and mitigating ROS improves the recovery of cells (Hong et al., 2019). This indicated that cells could accumulate sub-lethal damage which can be repaired depending on how they are allowed to recover; moreover, it points to a threshold of ROS exposure beyond which the *E. coli* cannot recover replicative capacity. According to one hypothesis, persisters that can replicate normally with the withdrawal of stress differ from the VBNC population that require special cultivation conditions for resuscitation because of the relative amount of protein damage they accumulate (Dewachter et al., 2021). In that study, growth to stationary phase caused depletion of ATP and induced protein aggregation, resulting in the formation of persisters and a VBNC population in *E. coli*. However, while persisters in general exhibited early developmental stage aggregates, the VBNC population had more mature aggregates. This phenomenon of “dormancy depth” has been explored in detail in another study demonstrating that the cell’s ability to disintegrate protein aggregates and restore proteostasis by recruiting DnaK-ClpB protein complexes to aggresomes is critical for its survival (Pu et al., 2019).

**BOX Overview of redox balance within mycobacterial cells**

** Redox stress and balance**

The redox environment of the cell is the sum total of the states of different redox couples as well as of the mechanisms which regulate the levels of these species, and disruptions to this environment can affect the culturability of Mtb (Sikri et al., 2018; Saito et al., 2021). More specifically, Sikri et al. reported the induction of stated Mtb upon treatment with vitamin C. This was accompanied by generation of non-cidal concentrations of H_2_O_2_, induction of ROS scavenging mechanisms, up-regulation of anabolic pathways of triacylglycerol (TAG) and sulfolipid (SL-1) synthesis. They also observed a reductive shift in intra-mycobacterial mycothiol redox potential upon vitamin C treatment in infected THP-1 cells. Saito et al., on the other hand, reported the formation of DD Mtb upon heat treatment, desiccation and starvation followed by rifampicin treatment. They further explored the mechanisms leading to the formation of DD Mtb and found production of ROS (superoxide), an oxidative shift in intra-mycobacterial mycothiol redox potential, and oxidative damage to major macromolecules and upregulation of oxidative stress response genes. Numerous studies have shed light on how the redox environment inside the cell affects Mtb’s response to various stress conditions like starvation, antibiotic treatment, acidic pH, hypoxia, nitrosative stress and during infection (Pacl et al., 2018; Mehta and Singh, 2019; Mishra et al., 2019; Mishra et al., 2021). Any imbalance in this environment can cause oxidative or reductive stress (Kumar et al., 2011). Oxidative stress is defined as an increase in the levels reactive oxygen species (ROS) over and above the mechanisms which detoxify them. Oxidative stress can be caused by diverse species of reactive oxygen molecules like superoxide radical ( ${\text{O}}_{2}^{•-}$ ), hydroxyl radical (HO^•^), hydrogen peroxide (H_2_O_2_), hydroxide ions (HO^−^), organic hydroperoxides (ROOH), peroxyl radical ( ${\text{RO}}_{2}^{•}$ ) and alkoxyl radical (RO^•^). Reductive stress results from the accumulation of reducing equivalents of NADH, NADPH, FADH_2_, MSH and EGH. These phenomena lie at the two extremes of the redox milieu. Increases in oxidative stress can lead to oxidative damage, affecting a cell’s ability to repair and replicate. On the other hand, increases in reductive stress can cause growth arrest, and an increase in drug tolerance and virulence phenotypes (Singh et al., 2009; Bhaskar et al., 2014; Trivedi et al., 2016; Mishra et al., 2019; Shee et al., 2022). In a recent study, Shee et al. showed that moxifloxacin treatment causes reductive stress in Mtb as displayed by the rise in NADH/NAD^+^ ratio, which in turn increases the labile, reduced form of Fe. This then fuels a Fenton reaction leading to ROS production. Addition of *N-*acetyl cysteine augmented ROS accumulation and apparent lethality when combined with moxifloxacin. High concentrations of cysteine had previously been shown to lead to increased levels of H_2_O_2_ (1.5- and 12-fold respectively for intracellular and extracellular H_2_O_2_) (Vilcheze et al., 2017). The combination of cysteine and isoniazid in that study shifted cells to a more reduced state with a higher menaquinol/menaquinone ratio and greater H_2_O_2_ levels (6- and 28-fold respectively for intracellular and extracellular H_2_O_2_) The role of reductive stress has also been studied in different aspects of Mtb biology (Trivedi et al., 2016; Coulson et al., 2017) and reviewed extensively elsewhere (Farhana et al., 2010; Mavi et al., 2020; Mishra et al., 2021; Singh et al., 2022). Thus, Mtb needs to sustain a delicate balance among different oxidizing and reducing species to maintain redox homeostasis.

**Protective mechanisms**

Mtb possess different mechanisms to deal with redox stress. These can be in the form of redox buffering systems like mycothiol, ergothioneine, thioredoxins and Dsb disulfide oxidoreductases. Mycothiol and ergothioneine are two low-molecular-weight thiols present in mycobacteria and they exist as oxidized–reduced redox couples (Cumming et al., 2018; Reyes et al., 2018). A mutant of mycothiol showed significantly more protein carbonylation and lipid peroxidation (Singh et al., 2016). Mtb also has thiol reductant proteins known as thioredoxins and Dsb disulfide oxidoreductases (Lu and Holmgren, 2014; Lin et al., 2016). Along with small molecules they are responsible for maintaining a reducing intracellular environment. Mtb also has antioxidant enzymes which can directly detoxify ROS, like catalase peroxidase (against H_2_O_2_), alkyl hydroperoxide reductases (against alkyl hydroperoxides) and superoxide dismutases (against ${\text{O}}_{2}^{•-}$ ). Increasing evidence has shown the non-conventional role of metabolic enzymes in countering oxidative stress, as seen with the alpha-ketoglutarate (alpha-KG) dehydrogenase complex (KDHC) and isocitrate lyase (Nandakumar et al., 2014; Maksymiuk et al., 2015). Redox mediated transcriptional regulators like DosT/DosS, whiBs (whiB3, whiB4, whiB7), PknG also play a key role in regulating pathways to counter the deleterious effect of damage caused by oxidative stress (Morris et al., 2005; Singh et al., 2007; Singh et al., 2009; Chawla et al., 2012; Mishra et al., 2017; Khan et al., 2017).

**Damage to macromolecules**

Oxidative stress can damage all classes of cellular macromolecules. Damage to DNA can be *via* oxidation of guanine leading to formation of 8-oxo-dG (OG) or oxidation of dCTP leading to DNA breakage (Foti et al., 2012; Vilcheze et al., 2013; Fan et al., 2018). Proteins can undergo oxidative modification which can affect their activity (Hillion and Antelmann, 2015; Hillion et al., 2017). Cysteine oxidation leads to the formation of sulfenic acids, sulfinic acid or sulfonic acid (Ezraty et al., 2017). Methionine can be oxidized to methionine sulfoxide, methionine sulfone or methionine sulfone. Proline, lysine, threonine, and arginine are prone to carbonylation (Nystrom, 2005). Cysteine, lysine, and histidine can also undergo carbonylation by reacting with carbonyl compounds on carbohydrates and lipids (Frank et al., 2002; Grimsrud et al., 2008). Irreversibly damaged proteins forms aggregates which cannot be degraded by proteasome and inhibits its activity. Oxidative damage to lipids can lead to the production of lipid peroxides affecting the function of cell membrane and cell wall where they are most abundantly found.

A similar observation was made in *Staphylococcus aureus* where host induced oxidative stress in the presence of antibiotics caused ATP depletion and induced formation of dormant states of persisters (Peyrusson et al., 2022). The degree of dormancy depth reached differed depending on the host cells, which induced in the bacteria different levels of oxidative stress. In high-oxidative-stress cells (for e.g., human macrophages, stimulated J774 macrophages, PMA-treated monocytes) ROS-induced ATP depletion caused higher protein aggregation, along with the recruitment of the DnaK-ClpB chaperone system. On the other hand, low-oxidative-stress cells (for example, untreated monocytes, unstimulated J774 macrophages epithelial cells, and osteoblasts) hosted a limited fraction of dormant persisters. The key difference between persisters isolated from low vs high-oxidative-stress cells was in the lag time before they resumed growth in liquid medium. Persisters from high-oxidative-stress cells took more time to regrow compared to those isolated from low-oxidative-stress cells, hypothesized to be either due to different levels of metabolism or the need for repair before regrowth. As in the *ex vivo* studies, time without replication appears to be a critical requirement for eventual growth in some phenotypes.

**Figure 3:**
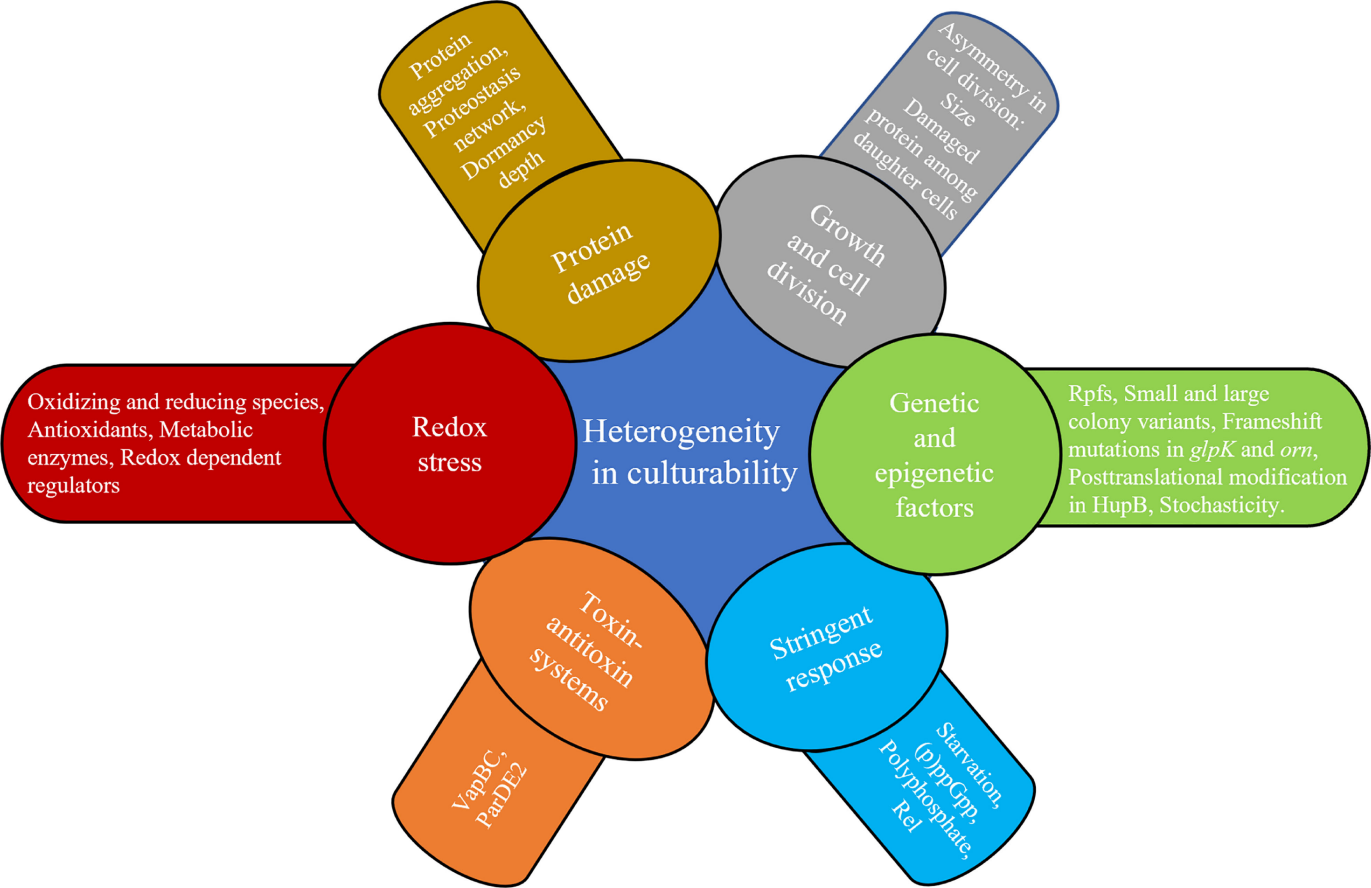
Factors that can introduce heterogeneity in culturability.

In mycobacteria, a relationship between exposure to stress and growth delay has been noted for decades. Studies in the 1960s found that Mtb exposed to TB drugs such as streptomycin and INH could induce days of delay in replication and, in the case of rifampin, with as little as 2 hours of exposure (Dickinson and Mitchison, 1970). In sputa from patients with TB, non-replicating Mtb containing lipid bodies have been found in patient sputa, and this phenotype was reproduced *in vitro* in response to hypoxic stress. In that study, the percentage of AFB with lipid bodies positively correlated with time to positivity in BACTEC liquid cultures (Garton et al., 2008). Automated image analysis of individual colonies from sputum samples of patients undergoing treatment for pulmonary TB found that persisters demonstrated longer lag times in colony formation compared with bacilli which are rapidly eliminated by TB therapy (Barr et al., 2016). Furthermore, counts of longer lag-time colonies (>20 days) declined more slowly than shorter lag-time colonies. *In vitro* work in *Mycobacterium smegmatis* (Msm) found that incubation in mild nutrient starvation or suboptimal growth media to stationary phase created small cells that displayed growth lag or non-culturability (Shleeva et al., 2004; Wu et al., 2016b). Similar findings were reported in Mtb that underwent gradual acidification in stationary phase, leading first to persistence, and then to an eventual DD Mtb state requiring Rpfs for resuscitation (Shleeva et al., 2011). A recent study from our group has shown Mtb can enter the DD state under diverse stresses, including with nutrient starvation followed by rifampin treatment, heat stress at 45°C or desiccation (Saito et al., 2021). We found that the formation of this population was linked to the presence of an intermediate amount of oxidative stress—rifampin exposure produced levels of ROS and an oxidative shift in cell state that were above other antibiotics that did not produce DD Mtb, such as levofloxacin, but below levels of a direct RNA polymerase inhibitor (a Nα-aroyl-N-aryl-phenylalaninamide compound) that apparently killed extensively and had no recoverable DD Mtb. *M. bovis* in the same conditions also suffered higher levels of oxidative stress than Mtb, and had no recoverable DD organisms. The results further suggested that a cell’s ability to prevent or repair that damage altered its culturability. Cultivating DD Mtb after serial dilution in liquid nutrient rich media created a longer lag time, and we speculate this time allowed the DD Mtb to repair damage and resume cell division (**Figure 2**). Prolonged incubation in nutrient-deficient PBS similarly prevented regrowth and allowed cells to reverse the levels of oxidative DNA damage to the pre-antibiotic exposure state. These cells eventually regained culturability even on agar plates. See **Table 1** for a summary of *in vitro* DD Mtb work; **Figure 3** highlights the different mechanisms influencing growth heterogeneity in Mtb. While promising, much work remains to determine how the *in vitro* studies relate to *in vivo* findings; discrepancies in, for example, antibiotic response kinetics between one *in vitro* model of persistence and clinical sputum have been noted (Faraj et al., 2020).

**Table 1:** *In vitro* and *ex vivo* studies of differentially detectable Mtb and DD Msm.

	*In vitro* or *Ex vivo*	Description of the study	Reference
1	*In vitro*	Mtb isolated from murine peritoneal macrophages	(Biketov et al., 2000)
2	*In vitro/In vivo*	Stationary phase Mtb incubated for 100 days in microaerophilic conditions then treated with rifampin; mice infected with Mtb, then treated with pyrazinamide and isoniazid	(Hu et al., 2000)
3	*In vitro*	In Mtb after growth in Sauton’s medium and prolonged incubation in stationary phase	(Shleeva et al., 2002)
4	*In vitro*	In Msm from stationary phase	(Shleeva et al., 2004)
5	*In vitro*	Wild type and the *rpf* deletion mutant Mtb strains aged in Sauton’s medium without oxygen	(Downing et al., 2005)
6	*In vitro*	In Msm after prolonged storage at room temperature in a nitrogen-limited minimal medium	(Anuchin et al., 2009)
7	*In vitro*	In Mtb after prolonged incubation in Sauton’s medium	(Salina et al., 2009)
8	*Ex vivo*	In sputum cultures of patients with TB before treatment, with and without Rpf supplementation	(Mukamolova et al., 2010)
9	*In vitro*	In Mtb after prolonged incubation in acidified Sauton’s medium	(Shleeva et al., 2011)
10	*Ex vivo*	Viable but non-cultivable Mtb in pulmonary and extra-pulmonary samples	(Cubero et al., 2013)
11	*Ex vivo*	In sputum cultures of patients with TB before and during early treatment, without Rpf supplementation	(Dhillon et al., 2014)
12	*In vitro*	In Mtb grown in potassium-deficient media	(Salina et al., 2014)
13	*In vitro*	In lungs of mice chronically infected with *Mtb*	(Manina et al., 2015)
14	*Ex vivo*	Mtb in sputum from patient samples	(Chengalroyen et al., 2016)
15	*Ex vivo*	In bioaerosols from untreated TB patients	(Patterson et al., 2017)
16	*In vitro*	In PBS starved, rifampicin treated Mtb	(Saito et al., 2017)
17	*Ex vivo*	In patient sputum before and during treatment in Haitian cohort	(McAulay et al., 2018)
18	*Ex vivo*	In extrapulmonary tuberculosis samples from patients recruited before the onset of chemotherapy	(Rosser et al., 2018)
19	*In vitro*	In Mtb exposed to vitamin C	(Sikri et al., 2018)
20	*In vitro*	In Mtb under lipid diet model	(Khan et al., 2019)
21	*In vitro*	Nitrite induces non-cultivability in Mtb	(Gample et al., 2019)
22	*In vitro*	Diphenyleneiodonium, an inhibitor of NADH oxidase, induced a viable, but non-culturable state in mycobacteria	(Yeware et al., 2019)
23	*In vitro*	In clinical isolates under Wayne’s model of hypoxia	(Dusthackeer et al., 2019)
24	*Ex vivo*	In pre- and post-treatment sputum samples from TB patients	(Almeida Junior et al., 2020)
25	*Ex vivo*	In induced sputum and bronchoalveolar lavage fluid from patients after therapy	(Beltran et al., 2020)
26	*Ex vivo*	Sputum from treatment naïve HIV-TB co-infected individuals	(McIvor et al., 2021)
27	*Ex vivo*	In the sputum of patients with drug-sensitive or drug-resistant TB	(Zainabadi et al., 2021)
28	*Ex vivo*	Sputum from individuals with drug susceptible TB	(Gordhan et al., 2021)
29	*Ex vivo*	Drug-Resistant Mtb in sputum from patients which only grew in glycerol-poor/lipid-rich medium	(Mesman et al., 2021)
30	*In vitro*	Mtb exposed to mild heat stress; Mtb desiccated on filters	(Saito et al., 2021)
31	*In vitro*	In Mtb from lung tissues derived from infected mice under specific drug treatments	(Evangelopoulos et al., 2022).

### Genetic and epigenetic factors

We have thus far noted the significant variabilities in mycobacterial growth between liquid and solid media, but bacilli can also demonstrate heterogeneity even as it grows as CFU. Generally, mycobacteria are known to grow as rough and dry, nonpigmented colonies. However, small colony variants (SCVs) and those with different colony morphologies can arise in response to host environments or drugs, as seen and studied in other bacteria such as *Staphylococcus aureus* (Proctor et al., 2006; Vulin et al., 2018). These morphologies can signal variability relevant to the behavior of the bacteria *in vivo*. Rough and smooth colony phenotypes have been identified in *Mycobacterium abscessus*, where the former stimulates the human macrophage innate immune response through TLR2, while the latter does not. This response is stimulated in part through phosphatidyl-myo-inositol mannosides which are present in both the variants but masked in the smooth variant by glycopeptidolipids present in their outermost portion of cell wall (Rhoades et al., 2009). This allows the smooth variant to restrict intraphagosomal acidification and induces less apoptosis and autophagy as compared to rough variant which induces the formation of autophagic vacuoles, rapidly acidification and apoptosis (Roux et al., 2016). The rough morphotype of *M. abscessus* forms clumps, unlike the smooth morphotype. Upon phagocytosis, the rough morphotype overwhelms the bactericidal capacities of J774 macrophages, killing them 72 hours after infection—while the macrophages infected with smooth morphotypes do not die even after 96 hours (Brambilla et al., 2016). The rough morphotype also produces proinflammatory cytokines and granuloma-like structures, while smooth morphotype does not. The rough colony morphotype of *Mycobacterium avium* also seems to be highly virulent in human macrophages and mice (Nishimura et al., 2020).

In Mtb, Safi et al. recently reported the presence of a sub-population of SCVs with a smooth morphology mixed with the large colony variants (LCVs) in clinical isolates of both drug sensitive and resistant Mtb strains. Whole genome sequencing of the SCVs identified frameshift mutations in *glpK* that disrupt its function. These mutations are reversible, and the mutants are tolerant to drugs and oxidative stress. They seem to represent a stress response which is activated when Mtb is starved for glycerol by either frameshifting *glpK* or by depleting glycerol from the medium (Safi et al., 2019). A similar mechanism of drug tolerance was found in reversible frameshift mutations in the Mtb *orn* gene which also produced SCVs (Safi et al., 2020). SCVs can also arise because of epigenetic regulation whereby posttranslational modification (lysine acetylation and methylation) of nucleoid-associated protein HupB results in a sub-population that exhibit heritable but semi-stable drug resistance (Sakatos et al., 2018).

While this review focuses on heterogeneity in growth in mycobacteria, and not phenotypic drug resistance per se, the interplay cannot be ignored. As previously mentioned, the mechanisms underlying the ability for persisters to survive drugs and their relationship to growth rate has been debated. Our discussion regarding the roles for stress and stress response in drug tolerance and growth rate point toward further questions that have been raised about these phenotypes. How much of the phenomenon is driven by direct damage to cellular processes, and how much is due to an adaptive response to that damage? (Nystrom, 2001) (Manina and McKinney, 2013) How much of any particular growth or drug resistance phenotype exists prior to a new stress in a stochastic manner, and how much is that subpopulation formed in response to the stress? How do the cells switch phenotypes (Dhar and McKinney, 2007)? Recent advances in microfluidics have shed light on some events which cause heterogeneity at the level of single cell (Santi et al., 2013). For example, using microfluidics, it was found that stochastic changes in the expression of catalase-peroxidase, *katG*, are negatively correlated with cell survival after INH treatment—but that growth rate prior to INH treatment was not correlated with the persistence phenotype (Wakamoto et al., 2013). Changes that cause drug tolerance among replicating mycobacterial subpopulations have also been described in bacilli subject to host induced stresses (Adams et al., 2011; Mishra et al., 2019). Adams et al. found that bacterial efflux pumps are responsible for the induction of drug tolerance in *Mycobacterium marinum* and Mtb during their replication inside zebrafish and macrophages, respectively. Mishra et al. extensively explored the role of redox heterogeneity in mediating drug tolerance in Mtb during infection. They found multiple mechanisms responsible for the induction of drug tolerance in a subpopulation exhibiting reduced mycothiol redox potential caused by phagosomal acidification. These included upregulation of genes encoding efflux pumps, oxidative stress response, DNA repair, protein quality control, envelope stress, sulfur metabolism, and SAM-dependent methyl transferases.

Single cell studies do, however, show growth rate diversity under *in vitro* growth conditions that is further amplified by stress conditions associated with infection and drug pressure (Manina et al., 2015). For example, a subpopulation of non-growing but metabolically active bacteria are found in chronically infected mice (Manina and McKinney, 2013; Manina et al., 2015). In another study, survival of mycobacterial cells upon exposure to ciprofloxacin was found to be correlated with the highly heterogenous rate of intermittent pulsing of *recA* expression—with increased pulsing prior to antibiotic exposure suggesting a response to greater spontaneous DNA damage. The work underscores the complexity of survival mechanisms and the influence of baseline phenotypic heterogeneity: highly pulsing cells grew at slower rates but lead to a higher likelihood of death upon exposure to drug, and yet overall the survivors exhibited significantly lower growth rates and 20% stalled replication entirely before drug exposure (Manina et al., 2019). The phenotypes of persistence cannot be binned based solely on growth parameters or stress response markers, and a nuanced approach is needed. Combining micro co-culture systems with single cell microfluidics may reveal more answers regarding bacterial behavior during unexplored aspects of host-pathogen interaction (Delince et al., 2016; Toniolo et al., 2018).

What seems clear now is that the mechanisms underlying stochastic, deterministic, and semi-heritable growth phenotypes—and their relationship to the ability to survive drug exposure—can be incredibly complex and specific. For example, Msm exposed to usually lethal doses of rifampin and observed by microscopy found multiple potential fates for any particular bacillus—not only could a cell have arrested growth, it could also continue to divide and yield daughter cells that were then either capable of division or not (Zhu et al., 2018). These growth phenotypes in the face of rifampin were dependent on individual accumulation of RpoB, the target of rifampin, after rifampin exposure. This, in turn, was determined by initial survival of rifampin exposure—perhaps by stochastic mechanisms—followed by upregulation of *rpoB* by the differential impact of rifampin on the two *rpoB* promoters. This particular mechanism of bacillary diversity in growth is thus specific to rifampin, has profoundly different effects on apparently genetically identical bacilli, and can affect subsequent generations.

### Asymmetric growth and division

Another source of heterogeneity on the single cell level is that mycobacteria grow and divide asymmetrically (Aldridge et al., 2012) (Joyce et al., 2012; Vijay et al., 2012; Vijay et al., 2014a; Vijay et al., 2014b; Rego et al., 2017; Priestman et al., 2017; Logsdon and Aldridge, 2018; Ufimtseva et al., 2019). The resultant cell size variability has been observed in *in vitro* models as well as in clinical samples (Vijay et al., 2017b; Ufimtseva et al., 2019; Pradhan et al., 2021). Differences in cell size yield differential susceptibilities to host and antibiotic stresses, which can in turn affect their survival and culturability (Richardson et al., 2016; Vijay et al., 2017a; Vijay et al., 2017b). For example, even log phase cultures of Msm and Mtb have been found to contain 2 sub-populations of cells which differ in size and density. Percoll density gradient centrifugation separated them into 2 distinct fractions—short-sized cells (SCs) and normal/long-sized cells (NCs). SCs were found to be more susceptible than NCs to antibiotics (rifampin and isoniazid), H_2_O_2_, and acidified NaNO_2_. Additionally, drug resistant bacteria display a distinct mode of cell division and cell length heterogeneity when compared with drug sensitive bacteria (Vijay et al., 2017b; Jakkala et al., 2020). Notably, mycobacteria exhibit a unique kind of heterogeneity under stress where it asymmetrically distributes irreversibly oxidized proteins within bacteria and between their progeny (Vaubourgeix et al., 2015). Another study done primarily in Msm was able to decrease the heterogeneity in cell size and growth rate by knocking out *lamA*, which was found to inhibit growth asymmetrically during the cell cycle (Rego et al., 2017). This in turn led to increased uniformity of killing by drugs that target the cell wall as well as less variability in growth rates in response to different concentrations of rifampin. These studies suggest that targeting mechanisms of heterogeneity may be vital to reducing treatment durations and improving relapse-free cure rates.

### Stringent response

The stringent response refers to a signaling system initiated by bacteria after encountering stress conditions (Irving et al., 2021). ppGpp (guanosine tetraphosphate) is an alarmone produced by bacteria which regulates the stringent response and is synthesized by the protein Rel in Mtb (Avarbock et al., 1999). Multiple studies have shed light on the role played by ppGpp in diverse mycobacterial species (Chakrabarty, 1998; Ojha et al., 2000; Primm et al., 2000; Manganelli, 2007; Wu et al., 2016a; Prusa et al., 2018; Bhaskar et al., 2018; Danchik et al., 2021; Hunt-Serracin et al., 2022). In other organisms, the stringent response is known to play an important role in the formation of the VBNC population. For e.g. *E. coli* mutants lacking ppGpp are less efficient in entering VBNC state compared to overproducers of ppGpp (Boaretti et al., 2003). Mutant of polyphosphate kinase 1 in *Campylobacter jejuni* deficient in polyphosphate was compromised in its ability to form VBNC (Ayrapetyan et al., 2015). Mutants lacking the *rel* gene were also unable to form a DD Mtb population in nutrient-starved Mtb exposed to rifampin, highlighting the role played by the stringent response in affecting culturability in mycobacteria (Saito et al., 2021).

### Toxin–antitoxin systems

Toxin-antitoxin (TA) systems have been identified in both bacteria and archaea (Gerdes et al., 2005; Yamaguchi et al., 2011), and play important roles in both physiology as well as pathogenesis. Mtb has more than 80 TA systems (Ramage et al., 2009; Sala et al., 2014) and many of them remain uncharacterized. TA systems have been implicated in the formation of a VBNC population in bacterial species like *E. coli* and *V. cholerae* (Ayrapetyan et al., 2015). In mycobacteria, transcriptome analysis of Mtb persisters showed upregulation of 10 TA modules (Keren et al., 2011). TA systems have also been implicated in the formation of a DD population in mycobacteria (Demidenok et al., 2014; Gupta et al., 2016). For example, overexpression of VapC toxin resulted in the production of ovoid cells which became non-culturable under potassium limiting conditions, while overexpression of VapB antitoxin prevented transition to this state. (Demidenok et al., 2014). Msm expressing the *parDE2* operon during oxidative stress also led to entry into the VBNC phenotype (Gupta et al., 2016). The same was found upon overexpression of parE2 toxin. Over-expression of MazF6 toxin resulted in a transcriptional profile which had significant overlap with the transcriptome of non-culturable cells (Ramirez et al., 2013). As we gain a better understanding of the role played by different TA systems in mycobacterial biology, future studies will reveal the mechanisms behind how they exert influence on culturability both *in vitro* and *in vivo*.

## Conclusion

The act of replication, while seemingly consistent and reproducible in a bulk, logarithmically-dividing mycobacterial culture, is heterogenous when examined more closely—and even more dynamic and variable in physiologic and artificially stressed conditions. This has profound clinical and research implications. In this review, we have scratched the surface of our increasingly nuanced understanding of growth phenotypes, as well as the strengths and limitations of our current culture-based tools to assess mycobacterial replication. Complexity further increases when deeply considering host-pathogen interactions in both clinical manifestations (e.g., in children, in people living with HIV, in patients who have received BCG vaccination, etc) and laboratory settings (e.g., animal and other infection models).

Heterogeneity in growth as has been described here is not specific to TB disease or even to infectious diseases, and many parallels have been drawn to cutting edge research in cancer (Glickman and Sawyers, 2012; Lupoli et al., 2018). For example, a recently published study of lung cancer cells found wide heterogeneity among the population that survived drug treatment, including a rare population of persisters that divided several times despite drug pressure. These “cycling” persisters demonstrated a strong link with antioxidant gene signatures and less ROS levels as compared to non-cycling brethren, and offers tantalizing insight into relapse and time to recurrence in cancer (Oren et al., 2021). Similar studies of mycobacterial population structure at the single cell level have revealed comparable complexity, and at a higher resolution than bulk culture methods allow—further refinement and application of these techniques may be critical to further clinical breakthroughs (Bussi and Gutierrez, 2019). Applying more granular assessment of mycobacteria as they move across stresses and transition between phenotypes may reveal significant clues as to how bacilli maintain or restore replicative capacity in the varied and harsh environments of its life cycle. This line of inquiry has a high likelihood of discovering mycobacterial vulnerabilities that may then be exploited clinically. That the infective dose_50_ is fewer than 10 bacilli mandates that a thorough understanding of TB infection and relapse cannot ignore the replication dynamics of even the smallest of subpopulations.

## Author contributions

KS and SM researched, wrote and edited the manuscript and figures. All authors contributed to the article and approved the submitted version.

## Funding

This work was supported by NIH grant K08 AI139360 to KS.

## Conflict of interest

The authors declare that the research was conducted in the absence of any commercial or financial relationships that could be construed as a potential conflict of interest.

## Publisher’s note

All claims expressed in this article are solely those of the authors and do not necessarily represent those of their affiliated organizations, or those of the publisher, the editors and the reviewers. Any product that may be evaluated in this article, or claim that may be made by its manufacturer, is not guaranteed or endorsed by the publisher.
